# An Analysis of Google Trends During COVID-19: Determining Public Urological Cancer Concerns

**DOI:** 10.7759/cureus.31752

**Published:** 2022-11-21

**Authors:** Fred Gong, Kyra Gassmann, Susan Gong, John Barlog, Andrew Winer

**Affiliations:** 1 Department of Urology, State University of New York (SUNY) Downstate Health Sciences University, Brooklyn, USA

**Keywords:** google trend, covid 19, diagnostic delay, delay treatment, urologic neoplasms

## Abstract

Background

The COVID-19 pandemic put a massive strain on the healthcare system as patients avoided the hospital, elective cases were postponed, and general medical anxiety was increased. We aimed to capture public interest in urological cancers during this massive shock to the medical field.

Methodology

A total of 12 keywords related to the three most prevalent urological cancers (prostate, bladder, and kidney) were searched using Google Trends from 2018 to 2022. The search volume index of these 12 keywords was extracted to assess public interest before and after the pandemic.

Results

There was a reduction in search volume for "prostate, bladder, and kidney cancer" and "kidney cancer treatment" after the postponement of elective surgeries. However, there was an increase in search volume for "prostate, bladder, and kidney cancer survival rates" and "prostate cancer symptoms" after this period. There was no change in search volume for bladder cancer symptoms, bladder cancer treatment, or kidney cancer symptoms.

Conclusions

Public interest in urological cancers decreased after COVID, while interest in survival rates across all three cancers increased. Future research is needed to investigate the effects of changing priorities and delays in medical care on patients’ experiences with urological cancers.

## Introduction

As COVID-19 became a global pandemic, new stay-at-home orders and delays in medical care resulted. Self-isolation, national lockdowns, and increased medical anxiety had the unintentional consequence of increased internet usage. Not only did the usage of internet services increase 40%-100% compared to pre-COVID-19 levels, but there was also an increasing public interest in COVID-19 in the United States [1,2]. In infodemiology, the science of determinants of information on the internet, data can be used to monitor internet interest in healthcare [3]. Previous urological studies have used infodemiology to determine that the internet interest in hematuria, kidney stones, and overactive bladder decreased during COVID-19 compared to pre-COVID-19 times [4].

Google Trends is a powerful tool that chronologically quantifies search interest on Google and accounts for over 80% of searches among platforms [5]. By providing insights into internet search trends and tracking all search queries made through the platform, Google Trends can be used for healthcare-related research. Data can be used to study internet health information−seeking behaviors and interest in various medical conditions within urology [6].

Urological conditions such as nocturnal enuresis, erectile dysfunction, and urological cancers affect people of all ages and are highly personal issues, which often result in the public using the internet to search for symptoms, treatment, and outcomes for potential conditions [7,8]. Additionally, there was much apprehension surrounding the impact of COVID-19 on urological conditions, which was propagated by social media, public fear [9], and delays in healthcare. The effect of delays in healthcare on urologists during COVID-19 has been documented as urologists dealt with increased anxiety, helplessness, and distress with a postponement in urological cancer care [10]. The purpose of this study was to use Google Trends, a major national search engine, to analyze internet search behaviors concerning the three most common urological cancers and their relation to the COVID-19 pandemic.

## Materials and methods

Our team queried Google Trends for search terms from the patient perspective after a urological cancer diagnosis. We worked with a urological oncologist to develop a list of search terms based on questions and concerns that patients commonly have. Our search terms list consisted of “Prostate Cancer,” “Prostate Cancer Survival Rate,” “Prostate Cancer Symptoms,” “Prostate Cancer Treatment,” “Kidney Cancer,” “Kidney Cancer Survival Rate,” “Kidney Cancer Symptoms,” “Kidney Cancer Treatment,” “Bladder Cancer,” “Bladder Cancer Survival Rate,” “Bladder Cancer Symptoms,” and “Bladder Cancer Treatment.” We chose to cover prostate cancer, kidney cancer, and bladder cancer as these are the three most common urological cancers [11]. Searches were limited to the USA between May 8, 2018, and January 21, 2022. Pre-COVID-19 was designated as May 8, 2018, to March 15, 2020 (677 days), and post-COVID-19 was designated as March 15, 2020, to January 21, 2022 (677 days). March 15, 2020, was chosen as the designated midpoint day because this was the week that the first stay-at-home orders were issued in the United States [12] and the week that the CMS recommended the postponement of elective procedures [13].

Data was reported from Google Trends as search volume index (SVI), a weighted scale from 0 to 100 of searches for specific terms relative to overall search volume. The weekly SVIs for all 12 search terms from May 8, 2018, to January 21, 2022, were used for data analysis. Pre-COVID-19 and post-COVID-19 median SVIs were compared using the Mann-Whitney U test. Subsequently, five subgroups of 24-week blocks were created to assess search trends as the pandemic progressed: 24 weeks pre-COVID-19, 0-24 weeks post-COVID-19, 24-48 weeks post-COVID-19, 48-72 weeks post-COVID-19, and 72-96 weeks post-COVID-19. The median SVIs of the four post-COVID-19 subgroups were each compared to that of the one pre-COVID-19 subgroup using the Mann-Whitney U test. SPSS version 28 was used to perform all statistics, with significance determined at *a *= 0.05.

## Results

**Figure 1:**
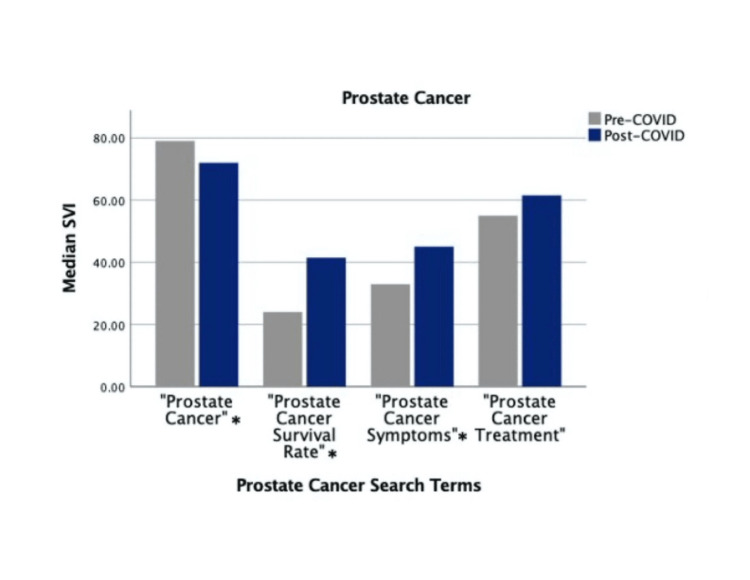
Pre-COVID-19 versus COVID-19 "Prostate Cancer" search volume. *Indicates statistically significant differences. SVI, search volume index

**Figure 2:**
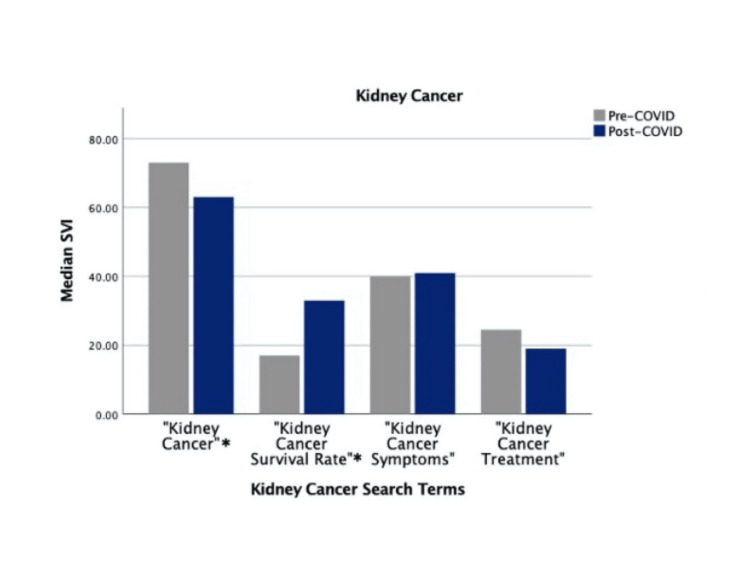
Pre-COVID-19 versus COVID-19 "Kidney Cancer" search volume. *Indicates statistically significant differences. SVI, search volume index

**Figure 3:**
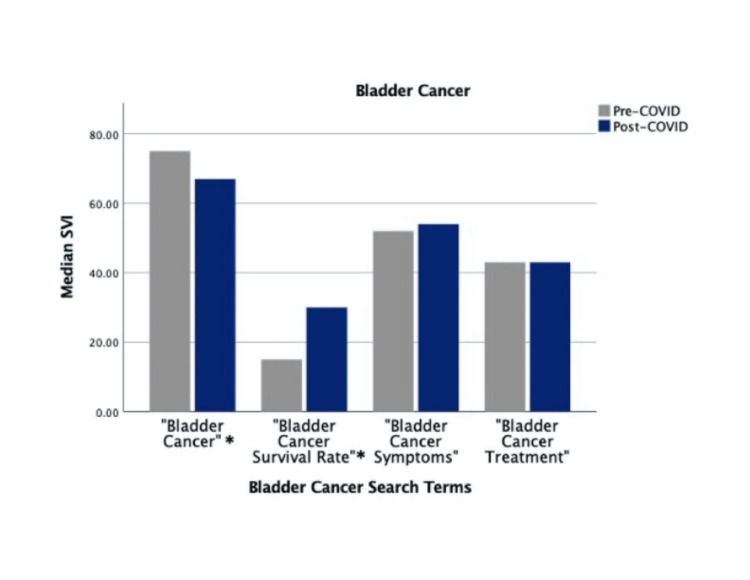
Pre-COVID-19 versus COVID-19 "Bladder Cancer" search volume. *Indicates statistically significant differences. SVI, search volume index

Search terms “Prostate Cancer Symptoms,” “Prostate Cancer Survival Rate,” “Kidney Cancer Survival Rate,” and “Bladder Cancer Survival Rate” showed significant increases in post-COVID-19 SVI (*P* < 0.001). Meanwhile, search terms “Prostate Cancer,” “Kidney Cancer,” “Kidney Cancer Treatment,” and “Bladder Cancer” showed significant decreases in post-COVID-19 SVI (*P *< 0.001). Search terms “Prostate Cancer Treatment,” “Kidney Cancer Symptoms,” “Bladder Cancer Symptoms,” and “Bladder Cancer Treatment” did not show significant differences in pre- and post-COVID-19 SVIs (Figures 1-3).

At 24 weeks post-COVID-19, 9 of 12 search terms showed decreased search volume compared to the pre-COVID-19 mark. At 48, 72, and 96 weeks post-COVID-19, only up to 2 of 12 search terms were searched more than at the pre-COVID-19 mark. “Kidney Cancer” was searched more during the pre-COVID-19 block than at 24, 48, 72, and 96 weeks post-COVID-19. “Prostate Cancer” was searched more pre-COVID-19 than at 24 and 48 weeks, with no differences at 72 and 96 weeks. “Bladder Cancer” was searched more pre-COVID-19 than at 24 and 96 weeks, with no differences at 48 and 72 weeks. “Prostate Cancer Survival Rate,” “Kidney Cancer Survival Rate,” and “Bladder Cancer Survival Rate” were all searched more pre-COVID-19 than at the 24 week post-COVID-19 mark. However, at 48, 72, and 96 weeks post-COVID-19, “Prostate Cancer Survival Rate” was searched more than during the 24 weeks preceding COVID-19. “Kidney Cancer Survival Rate” and “Bladder Cancer Survival Rate” were both searched more at 48 and 72 weeks post-COVID-19 than at 24 weeks pre-COVID-19. “Prostate Cancer Symptoms” followed a similar trend with higher pre-COVID-19 search volume when compared to 24 weeks post-COVID-19 while having higher search volume at 72 and 96 weeks post-COVID-19. The phrase “Prostate Cancer Treatment” was searched more at 24, 72, and 96 weeks post-COVID-19; however, it did not have significantly higher search volume over the entire 96-week time frame (Table 1).

**Table 1:** Comparison of 24-week subgroups *Significant decrease in search volume <0.05. **Significant increase in search volume <0.05. SVI, search volume index

	Search term	Pre-COVID-19 median SVI (-24 weeks to 0 weeks)	0-24 weeks post-COVID-19 median SVI (*P*-value)	24-48 weeks post-COVID-19 median SVI (*P*-value)	48-72 weeks post-COVID-19 median SVI (*P*-value)	72-96 weeks post-COVID-19 median SVI (*P*-value)
Prostate cancer	Prostate cancer	79.5	63 (<0.001)*	74.5 (0.017)*	75 (0.12)	76 (0.079)
	Prostate cancer symptoms	38	33 (0.06)	42.5 (0.54)	47.5 (0.002)**	57 (<0.001)**
	Prostate cancer survival rate	22.5	13.5 (0.033)*	46 (<0.001)**	48 (<0.001)**	49 (<0.001)**
	Prostate cancer treatment	56	58.5 (<0.001)**	56.5 (0.77)	67.5 (0.027)**	67 (0.027)**
Kidney cancer	Kidney cancer	74	59.5 (<0.001)*	64.5 (0.002)*	67 (0.005)*	58 (<0.001)*
	Kidney cancer symptoms	44	35 (0.049)*	57 (0.015)**	44 (0.79)	39.5 (0.44)
	Kidney cancer survival rate	23	14 (0.12)	32.5 (0.009)**	49 (<0.001)**	29.5 (0.20)
	Kidney cancer treatment	23	18.5 (0.20)	21.5 (0.61)	23.5 (0.91)	17 (0.36)
Bladder cancer	Bladder cancer	73	58.5 (<0.001)*	70 (0.13)	75.5 (0.50)	65.5 (<0.001)*
	Bladder cancer symptoms	56.5	33.5 (<0.001)*	63.5 (0.34)	66.5 (0.14)	56 (0.98)
	Bladder cancer survival rate	12.5	6 (0.031)*	19.5 (0.027)**	21 (0.016)**	15 (0.17)
	Bladder cancer treatment	74	42.5 (0.59)	34.5 (0.19)	49.5 (0.10)	50.5 (0.19)

## Discussion

In an age when medical information is easily accessible on the internet, Google Trends has proven to be a useful tool for healthcare providers to assess the public’s interest in specific health-related topics by providing information on the search volumes of specific terms [14]. Google Trends has been utilized throughout the field of urology to analyze internet interest in topics such as kidney stone surgery and reconstructive urology [15,16]. However, there are a few studies that analyze the effect of COVID-19 on urological cancers. The goal of our study was to take a look into how the COVID-19 pandemic influenced internet interest related to the three most common urological cancers: kidney, prostate, and bladder.

Our study results demonstrated that there was a statistically significant decrease in the search volume for the terms “Bladder Cancer,” “Kidney Cancer,” and “Prostate Cancer” in the post-COVID-19 era. Khene et al. found similar decreased interest in the aforementioned terms in their Google Trends study analyzing the impact of COVID-19 on internet interest in urological cancers [17]. Specifically, at 24 weeks post-COVID-19, 9 of 12 possible search terms showed decreased post-COVID-19 search volume. We interpreted these results to reflect a heightened focus on COVID-19 causing patients’ attention to be taken away from other health issues, leading to a decreased number of searches related to these urological conditions. Additionally, with less frequent in-person visits to the doctor during the pandemic [18], there was likely underdiagnosis of these urological cancers, leading to less internet interest. This is in concordance with a study by London et al. that looked at how the pandemic affected the number of cancer-related patient encounters across the United States [19]. They found that there was a 57% decrease in cancer-related visits and a 74% decrease in new incidence cancer visits when comparing April 2020 to April 2019 [19]. This indicates not only a decrease in cancer patients attending their appointments but also in the diagnosis of new cancers. This underdiagnosis can also potentially explain our finding of a decrease in SVI for “Kidney Cancer Treatment” in the post-COVID-19 period when compared to the pre-COVID-19 period. With fewer new cancer diagnoses, it can be expected that searches surrounding cancer treatment are decreased. Underdiagnosis and delayed treatment of urological cancers are of major concern as delays in cancer management may lead to worse outcomes. Delays of more than six months in clinically diagnosed T2 renal cancers result in worse overall survival [20], and delays in cystectomy in bladder cancer lead to worse outcomes and prognosis [21]. Radical prostatectomy in prostate cancer patients, even in those with high-risk disease, can be safely delayed up to six months [22]. However, the effects of longer postponement of treatment are unclear, especially regarding the underdiagnosis of disease.

There was a statistically significant increase in searches for the terms “Kidney Cancer Survival Rate,” “Prostate Cancer Survival Rate,” and “Bladder Cancer Survival Rate’’ in the post-COVID-19 period. This finding suggests that although general interest in urological cancers decreased, anxiety regarding the prognosis of urological cancers increased. At the start of the COVID-19 pandemic, Stensland et al. proposed guidelines for triaging urological surgeries in the setting of limited resources due to COVID-19 [23]. They recommended that most prostatectomies, even in some cases of high-risk disease, and kidney resection for stages I and II tumors be delayed to prioritize more urgent cases [23]. Adoption of guidelines such as this likely sparked anxiety in cancer patients whose treatments were not able to be delivered as timely as in the pre-COVID-19 era. Interestingly, there was an initial decrease in search volume for urological cancers’ survival rates at 24 weeks post-COVID-19. However, search volume for survival rates was, for the most part, higher at the 48-, 72-, and 96-week post-COVID-19 marks. This trend could reflect the initial shock of COVID-19 followed by a rebound in medical anxiety. Our Google Trends analysis also revealed a significant increase in SVI for “Prostate Cancer Symptoms” (*P* < 0.001) post-COVID-19. This could be due to prostate cancer patients being disproportionately affected by surgical delays and, thus, has increased concern about their cancer progression, leading to more Google searches. Mian et al. surveyed urological oncologists to get firsthand information about the delays in urological cancer treatment during the COVID-19 pandemic. They found that the most significant disruption in urological surgeries occurred with prostate cancer, with 59% of respondents reporting that all surgeries for prostate cancer had been postponed where they practice [24].

This study is not without limitations. First, our team only looked at Google to collect our data for internet search volume. Although Google is the most commonly used search engine, accounting for over 80% of search engine foot traffic [5], other sites such as Bing and Yahoo were not taken into account. Furthermore, social media has become a popular place to seek out health information [25]. Looking at searches on social media platforms, such as Facebook and Twitter, could have led to more robust data. Additionally, Google Trends has no way of providing us with the demographics of those conducting the searches, and therefore, there is no way of being certain that the increased volume of searches was due solely to patient activity, as providers or families may have been conducting their own internet searches as well. While we can infer motives behind changes in search volumes for specific terms, we cannot completely confirm these motives without a formal survey of patients and their sentiments. Future research must be done to investigate the etiologies causing differences in interest in urological cancers and the effects of COVID-19 on both urological patients and providers.

## Conclusions

In conclusion, internet interest in prostate, kidney, and bladder cancers’ survival rates significantly increased during COVID-19 compared to pre-COVID-19. Delayed treatment and postponement of elective surgeries and procedures may have increased interest in survival rates. Specifically in prostate cancer, the increased search volume in prostate cancer symptoms and survival rate may be due to the higher rates of postponements of treatment and elective surgeries compared to both bladder cancer and kidney cancer. Meanwhile, general internet interest in these cancers decreased. This decreased search volume may indicate that there was less interest in urological oncology health, which may have been due to fewer doctors’ visits and fewer diagnoses during this time. Overall, we hope that this information can inform the public on internet interest in urological cancers and help guide future research into patient and caregiver urological cancer anxiety.
